# Individual Alpha Peak Frequency in Ice Hockey Shooting Performance

**DOI:** 10.3389/fpsyg.2017.00762

**Published:** 2017-05-16

**Authors:** Sommer Christie, Selenia di Fronso, Maurizio Bertollo, Penny Werthner

**Affiliations:** ^1^Faculty of Kinesiology, University of Calgary, CalgaryAB, Canada; ^2^Behavioral Imaging and Neural Dynamics Center, Department of Medicine and Aging Sciences, G. d’Annunzio University of Chieti-PescaraChieti, Italy

**Keywords:** EEG, individual alpha peak frequency, ice hockey, performance, Psychophysiology

## Abstract

There are several important inter- and intra-individual variations in individual alpha peak frequency (IAPF) in the cognitive domain. The rationale for the present study was to extend the research on IAPF in the cognitive domain to IAPF in the sport domain. Specifically, the purpose of the present study was twofold: (a) to explore whether baseline IAPF is related to performance in an ice hockey shooting task and (b) to explore whether a shooting task has an effect on IAPF variability. The present investigation is one of the first studies to examine links between IAPF and sport performance. Study results did not show significant changes in IAPF when comparing baseline IAPF and pre- to post-task IAPF across three performance levels. The findings support previous literature in the cognitive domain suggesting that IAPF is a stable neurophysiological marker. Future research should consider the following methodological suggestions: (a) measuring IAPF during sport performance instead of at a resting state, (b) changing the pre-performance resting baseline instructions to take into account sport-specific mental preparation, (c) exploring an expert-novice paradigm to accentuate performance ability differences between groups (d) comparing tasks with different levels of complexity, and (e) analyzing the possible correlation between IAPF and performance on different days.

## Introduction

Alpha-band oscillations are the most dominant frequency in the adult human brain with a mean frequency of approximately 10 Hz and a range in oscillation of 7.5–12.5 Hz (Klimesch, 1999, 2012). Cheron et al. (2016) have recently suggested alpha oscillations as one of the five Electroencephalography (EEG) biomarkers, useful for exploring brain activity in sport. In particular, alpha oscillations reflect global resting state, cognitive performance, selective attention, inhibition, and gating. Alpha amplitude is at its peak in an eyes-closed resting condition primarily due to lack of sensory input. Suppression of alpha amplitude occurs in response to eye opening (Berger, 1929). Alpha is manifested by a ‘peak’ in the spectral analysis that can be used as a distinct marker to delineate the upper from the lower alpha band (e.g., Doppelmayr et al., 1998; Klimesch, 1999). Individual alpha peak frequency (IAPF) is defined as the maximum power value in the EEG frequency spectrum between 7.5 and 12.5 Hz (Klimesch, 1999). For healthy adults IAPF lies between 9.5 and 11.5 Hz (Klimesch, 1999). Research exploring IAPF has identified several important inter- and intra-individual differences (e.g., Angelakis et al., 2004a; Haegens et al., 2014). Inter-individual differences have been attributed to genetic factors (e.g., Smit et al., 2006; Bodenmann et al., 2009) and age suggesting that IAPF increases until puberty and declines after 40 years of age (Bazanova and Aftanas, 2008; Berchicci et al., 2011). In addition, lower IAPF is found in individuals with various brain pathologies including traumatic brain injury (TBI) (Angelakis et al., 2004a), chronic fatigue syndrome (Billiot et al., 1997), schizophrenia (Canive et al., 1998), stroke (Juhasz et al., 1997), Alzheimer’s disease (Klimesch et al., 1990), attention deficit hyperactivity disorder (Lansbergen et al., 2011), and major depression (Arns et al., 2012). In healthy subjects, higher IAPF has been correlated with good memory performers (Klimesch et al., 1993), better working memory scores (Grandy et al., 2013a), speed of information processing (Bornkessel et al., 2004), and shorter visual reaction times (Jin et al., 2006).

Higher IAPF has been suggested to reflect a state of ‘cognitive preparedness’ defined by Angelakis et al. (2004a) as a measure of the brain’s capacity for cognitive function and “refers to a brain trait or state that sets the stage for optimal cognitive performance” (p. 889). Thus, not only is IAPF a stable neurophysiological ‘trait’ marker (Grandy et al., 2013a) but it can also be seen as a ‘state’ variable that demonstrates intra-individual variability (Haegens et al., 2014). As suggested by Angelakis et al. (2004b) inter-subject variability or ‘trait’ IAPF is best mirrored during pre-performance rest periods and that intra-subject variability or ‘state’ IAPF is best reflected in the difference between pre- and post-task rest periods.

Research exploring intra-individual differences in IAPF has begun to highlight several critical factors in relation to the effect of different types of performance (cognitive and physical) on the brain capacity. In a two-part study, Angelakis et al. (2004a) demonstrated that subjects with TBI had significantly lower IAPF compared to healthy controls (matched for age, sex, and education level) in post-task eyes-open resting condition supporting prior research where IAPF reflects a ‘trait’ of cognitive preparedness. In the second experiment, testing ‘state’ cognitive preparedness, the authors found that in non-clinical subjects pre-task IAPF (eyes-closed baseline recording) was significantly correlated with working memory performance on the 1st day of testing but not on the 2nd day. This research suggests that pre-task IAPF may fluctuate for individuals and reflect the moment-to-moment readiness of the individual to perform (cognitive preparedness). The authors also noted that there was no significant correlation for post-test IAPF and performance. When groups were separated into high-IAPF and low-IAPF groups based on median performance score, they found that IAPF increased significantly for individuals with low-IAPF and did not for those with high-IAPF pre-task, suggesting that those with low-IAPF may not have been as “prepared” for the cognitive task and therefore increased their IAPF to compensate for this lack of preparedness. Finally, both pre-task IAPF and performance on day two was significantly higher than on day one suggesting that part of the ability to perform better in a task is related to cognitive preparedness. Similar to the above findings, Haegens et al. (2014) demonstrated that an increase in IAPF (positive ‘shift’) occurs during challenging cognitive tasks suggesting that this increase in IAPF could be indicative of either the activation of different groups of neurons (Sadaghiani et al., 2010) or reflective of active engagement of the system due to increased cognitive demand.

Some studies have begun to extend the findings from the cognitive domain and apply those findings to a sensorimotor domain during actual performance. For example, one study found that a positive shift in IAPF was associated with increasingly difficult balancing tasks suggesting that the shift is related to an “increase in cortical resource investment and activation” (Hülsdünker et al., 2015, p. 9). Another study, by Gutmann et al. (2015), examined the relationship between state variability of IAPF and physical activity. They found that IAPF increased significantly after exhaustive exercise but not after steady state exercise, suggesting the increase in IAPF reflects an increasing level of preparedness for external input and a higher level of arousal, which in turn, may facilitate information processing.

Research with athletes has used the IAPF to calculate individual alpha bandwidths (i.e., Del Percio et al., 2009; Babiloni et al., 2010), however, the research has not extensively explored changes in IAPF due to engagement in a sport related task or the potential relationship between IAPF and sport performance. The ability to focus attention, process information, and react quickly are all skills essential in achieving optimal performance in sport. Therefore exploration of the potential link between the qualities associated with higher IAPF such as cognitive preparedness (Angelakis et al., 2004a), speed of information processing (Bornkessel et al., 2004), and shorter visual reaction times (Jin et al., 2006), and sport performance is a critical step in the exploration of IAPF and optimal sport performance. Thus, the purpose of the present study was to extend previous research on IAPF in the cognitive domain and address it in the sport domain. Similar to suggestions by Angelakis et al. (2004a) and Gutmann et al. (2015) it was hypothesized that (a) the baseline IAPF will reflect a level of cognitive preparedness linked to the level of performance in a sport task, and (b) an increase in IAPF pre- to post-sport performance will be related to the level of psychomotor demand specific to the sport task.

## Materials and Methods

### Participants

Twenty-eight university level ice hockey players (15 female, 13 male; mean age 21.8 ± 2.0 years; range 19–27 years) participated in this study. The athlete-participants were all currently playing for university ice hockey teams. Ethical approval for this research was obtained from the primary author’s University Health Research Ethics Board. After being briefed on the overall purpose of the research study, the athlete participants signed a written informed consent form. Two subjects were eliminated from the final analysis due to faulty EEG recording at baseline.

### Procedure

Initially, 120 s of eyes-closed resting EEG followed by 120 s of eyes-open resting EEG was recorded. Immediately after, athlete-participants completed 25 shots on net, followed by a short rest period of 30 s (to simulate time on bench between shifts) and another 25 shots on net. Finally, immediately after the second set of shots on net, 120 s of eyes-open EEG was recorded in a final seated resting state. Although optimal experimental conditions for determination of IAPF are eyes-closed condition (e.g., Klimesch et al., 1993; Bazanova and Vernon, 2014), recordings with eyes-open were chosen to provide a more ecologically valid representation of a hockey-related task similar to other recent research conducted comparing IAPF at rest and during tasks (i.e., Haegens et al., 2014).

As suggested by Angelakis et al. (2004a), inter-subject variability or ‘trait’ IAPF is best mirrored during pre-performance rest periods and that ‘state’ IAPF or intra-subject variability is best reflected in the difference between pre- and post-task rest periods. Therefore, in the present study, IAPF was calculated at two time points for each individual in order to determine trait and state variability related to an ice hockey shooting performance task. A comparison was then conducted between three groups (top, moderate, and bottom performers) to explore the differences in IAPF and its link to performance (number of successful shots on net).

### Ice Hockey Shooting Performance Measure

**FIGURE 1:**
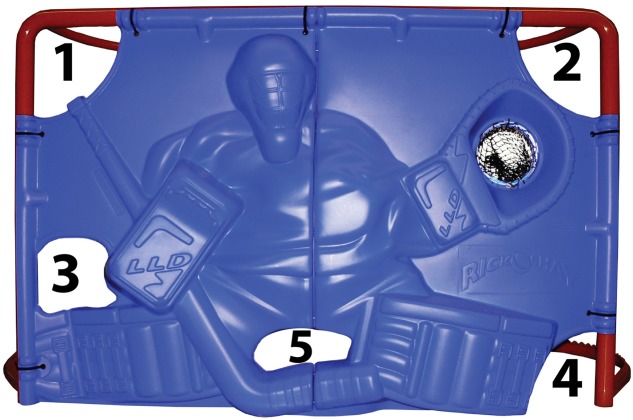
**Target 1 = stick, side high, target 2 = glove side high, target, 3 = stick side low, target 4 = glove side low, and target 5 = five hole (Rick-O-Shay hockey goal blocker)**.

The ice hockey shooting performance protocol was developed with the assistance of Hockey Canada’s Vice President and Manager of Player Development, and was designed to reflect, as much as possible, ecologically valid shooting performance conditions. Further, intra-class correlation coefficients (ICC) and Cronbach Alpha were used to assess reliability between sets of five baseline shooting performance scores. A high degree of reliability was found between shooting performance measurements. The ICC was 0.899 with a 95% confidence interval from 0.829 to 0.947 and the Cronbach’s alpha was 0.916. Ice hockey shooting performance was measured as successful number of shots on net out of 50. Athlete-participants completed a total of 50 shots on net taken on five possible randomized targets (**Figure 1**). Athlete-participants were wearing their own hockey skates and gloves to simulate actual shooting conditions. The athletes stood on synthetic ice approximately 16 feet away from the net for shooting. Affixed behind each target was a light that flashed either as a warning signal or a signal to shoot at that target. The shooting protocol began with a warning signal where all five lights illuminated in quick succession, followed by the signal light that illuminated in one of the five target locations (random location, random time) indicating to the athlete to shoot at that target. If the puck goes in the net it is counted as a successful shot and if the puck does not go in the net it is counted as not successful.

### EEG Data Acquisition

Electroencephalography data was continuously recorded at 19-sites using the eego^TM^ sports 64 pro EEG system with ANT EEG electrode array WaveGuard caps that allow for quality EEG measurement during movement (Advanced Neuro Technology, Enschede, Netherlands). Nineteen electrodes were chosen: F7, F3, Fz, F4, F8, M1, T7, C3, Cz, C4, T8, M2, P7, P3, Pz, P4, P8, O1, and O2. The choice of electrode sites was made in order to ensure the time-consuming setup and recording of EEG for multiple athletes per day was feasible. The 19 electrodes were placed in accordance with the International 10/20system (Jasper, 1958). EEG recordings were acquired at a sampling frequency of 500 Hz and recorded with a common reference. Ground electrode was located at AFz and impedance values were below 10 KΩ.

### EEG Analysis

Electroencephalography data were analyzed using ASAlab^TM^ software system. A band pass filter between 0.3 and 40 Hz was applied to the EEG data. EEG data was visually inspected to identify artifacts (instrumental, ocular, and muscular) and the artifact correction feature in ASAlab^TM^ software (Zanow and Knösche, 2004) was used to correct them. Any channels or segments with residual artifact were excluded from further analysis.

Individual alpha peak frequency identification was conducted by exploring source-level power spectra at two occipital (O1 and O2) and five parietal (P7, P3, Pz, P4, and P8) sites using a fast Fourier transform (FFT). These sites were selected based on the work of Bazanova (2011) suggesting that the best location to identify the frequency range of IAPF is located in the posterior regions of the brain. IAPF was identified as the center of gravity frequency within the 7–14 Hz band. The center of gravity frequency refers to the “weighted sum of spectral estimates divided by alpha power”:

$${\frac{\Sigma \left(a(f)\times f\right)}{\left(\Sigma a\left(f\right)\right)}}_{\left(K\,\lim esh,\;1999,\;p.\,\;171\right)}$$

This approach was chosen over visual inspection of the highest local peak in the spectra in order to be sensitive to the entire shape of the alpha peak (Klimesch et al., 1993). Furthermore, the gravity frequency approach has been suggested to provide a more accurate estimate of alpha frequency particularly in cases when multiple peaks are identifiable (Klimesch, 1999). A wide alpha band (7–14 Hz) was chosen to explore IAPF because it has been shown that alpha rhythms operate across a wider frequency than traditional narrow fixed bandwidths (i.e., 8–12 Hz) and this may lead to missing IAPF that lies outside of the traditional fixed bandwidths (Haegens et al., 2014). If no peak was present, it was not counted.

### Statistical Analysis

For statistical analysis, two participants (numbers 17 and 22) were eliminated because no visible alpha peak was present. Therefore, twenty-six athlete-participants (*n* = 13 female, *n* = 13 male) were divided into tertiles according to the combination of gender and shooting performance score. Females scored (16.23 ± 5.23) significantly lower (*p* < 0.005) than males (22.23 ± 4.97) on the ice hockey shooting performance test, therefore, females and males were separated into tertiles independently by a performance score out of 50 and then placed into the three performance groups. The three groups included; Group 1: top performers (*n* = 8), Group 2: moderate performers (*n* = 6), and Group 3: bottom performers (*n* = 12). Descriptive statistics for each participant is shown in **Table 1**. The comparison of groups was chosen for analysis based on previous research exploring differences in baseline IAPF and its relationship to performance (e.g., Angelakis et al., 2004a). Chi-squared analysis demonstrated no gender bias among these three groups [χ^2^(2) = 1, *p* = 0.607]. One-way ANOVA (score by group) demonstrated that the groups were significantly different [*F*_(2,23)_ = 14.056, *p* < 0.001]. Top performers scored 25.38 ± 4.2 out of 50 shots, moderate performers scored 18.5 ± 4.09 out of 50 shots, and bottom performers scored 15.5 ± 4.03 out of 50 shots. *Post hoc* analysis (Tamhane) revealed that there was no significant difference (*p* = 0.431) between the moderate performers (group 2) and the bottom performers (group 3).

**Table 1:** Individual values.

Athlete	Baseline performance score/50	IAPF eyes-closed baseline (Hz)	IAPF eyes-open baseline (Hz)	IAPF eyes-open post-task (Hz)	Shift in IAPF eyes-open pre- to post-task (Hz)
1	20	10.36	9.85	10.01	0.16
2	26	9.91	10.02	9.82	-0.20
3	13	10.73	10.37	10.31	-0.06
4	10	10.21	10.38	10.31	-0.07
5	22	9.09	9.50	9.56	0.06
6	23	10.79	10.29	10.21	-0.08
7	16	9.78	10.06	10.3	0.24
8	26	9.99	9.79	10.06	0.27
9	29	10.59	10.55	10.31	-0.24
10	17	9.66	10.00	9.91	-0.09
11	14	10.42	10.03	9.99	-0.04
12	19	9.97	10.18	10.21	0.03
13	32	9.78	10.21	10.34	0.13
14	20	9.86	10.09	10.12	0.03
15	17	10.50	9.33	10.04	0.71
16	15	10.25	10.03	10.04	0.01
17	17	*Eliminated no peak present*			
18	18	10.79	10.05	10.05	0.00
19	20	9.94	9.68	10.01	0.33
20	25	10.04	10.16	10.26	0.10
21	15	9.35	9.45	9.37	-0.08
22	8	*Eliminated no peak present*
23	9	10.92	10.21	10.35	0.14
24	26	9.75	10.16	10.26	0.10
25	23	9.98	10.19	10.1	-0.09
26	17	10.11	10.51	10.29	-0.22
27	11	10.12	9.98	9.85	-0.13
28	17	9.93	10.01	9.64	-0.37
	**18.75 ± 6.03**	**10.11 ± 0.44**	**10.04 ± 0.30**	**10.07 ± 0.26**	**0.025 ± 0.21**


Baseline IAPF (parietal and occipital average) was compared in eyes-closed resting condition between groups using a one-way ANOVA with factors group (top, moderate, and bottom performers).

The shift in IAPF from eyes-open pre- and post-shooting task was completed using a two way mixed ANOVA with one between subject factor, namely, group of 3 levels (top, moderate, and bottom performers), and one within subject factor, namely time of two levels (pre- and post- shooting task).

Angelakis et al. (2004a) demonstrated IAPF increased more for individuals with low peak alpha frequency (low-PAF) than subjects with high peak alpha frequency (high-PAF) suggesting that those with low peak alpha frequency were not fully prepared for the task and “were forced by the task to correct their unpreparedness by increasing their PAF” (Angelakis et al., 2004a, p. 894). Drawing on this research, further analysis of the shift in IAPF was conducted by splitting participants into two groups: those with an IAPF that was higher than the group’s median at resting baseline (eyes-open; high-IAPF, *n* = 13), and those with IAPF that was lower than the group’s median at resting baseline (eyes-open; low-IAPF, *n* = 13). The high-IAPF group had a mean alpha peak frequency (APF) of 10.23 ± 0.15 Hz and the low-IAPF group had a mean APF of 9.82 ± 0.25 Hz. Chi-squared analysis demonstrated no gender bias among these three groups [χ^2^(1) = 1, *p* = 0.239]. The shift in APF for high- and low-IAPF groups was completed using a two way mixed ANOVA with one between subject factor, namely, group of two levels (high- and low-IAPF), and one within subject factor, namely time of two levels (pre- and post- shooting task).

## Results

### Baseline IAPF and Ice Hockey Shooting Performance

**FIGURE 2:**
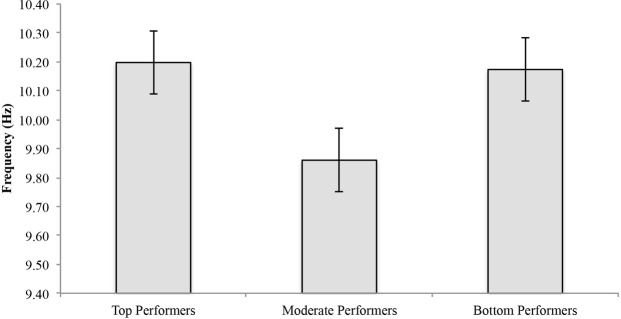
**Individual alpha peak frequency by performance group (top, moderate, and bottom) in eyes-closed baseline resting condition**.

In eyes-closed resting conditions pre-shooting task, the mean APF for top performers was 10.2 ± 0.45 Hz, 9.86 ± 0.54 Hz for moderate performers, and 10.17 ± 0.38 Hz for bottom performers (**Figure 2**). No significant difference [*F*_(2,23)_ = 1.255, *p* = 0.304] among groups was found in eyes-closed resting condition pre-shooting task. See **Table 1** for individual values.

### Shift in IAPF in Ice Hockey Shooting Task: Top, Moderate, and Bottom Performance Group Comparison

**FIGURE 3:**
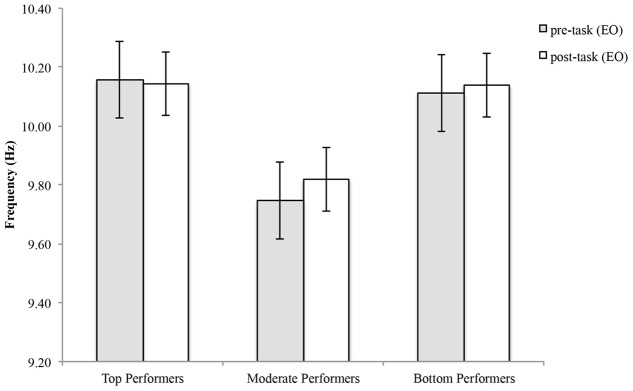
**Alpha peak frequency pre-and post-shooting performance for top, moderate and bottom performers**.

For top performers, mean APF decreased an average of 0.0138 ± 0.173 Hz and mean APF increased an average of 0.0717 ± 0.355 Hz for moderate performers and 0.027 ± 0.162 Hz for bottom performers (**Figure 3**). See **Table 1** for individual values. Statistical analysis showed a significant group effect [*F*_(2,23)_ = 6.11, *p* = 0.007], however, there was no statistically significant time by group interaction effect [*F*_(2,23)_ = 0.256, *p* = 0.777], and no statistically significant time effect [*F*_(1,23)_ = 0.389, *p* = 0.539].

### Shift in IAPF in Ice Hockey Shooting Task: High- and Low-IAPF Group Comparison

**FIGURE 4:**
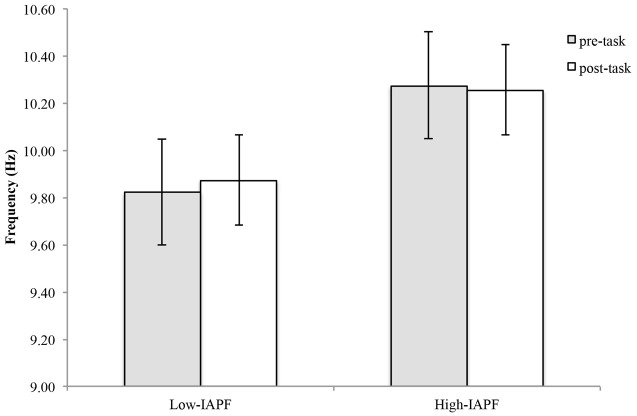
**Alpha peak frequency pre-and post-shooting performance for high- and low-IAPF groups**.

For the high-IAPF group, APF decreased by an average of 0.02 ± 0.13 Hz and increased an average of 0.06 ± 0.27 Hz for the low-IAPF group (**Figure 4**). Statistical analysis showed a statistically significant group effect [*F*_(1,24)_ = 45.88, *p* < 0.001], however, there was no statistically significant time by group interaction effect [*F*_(1,24)_ = 0.311, *p* = 0.582], and no statistically significant time effect [*F*_(1,24)_ = 0.332, *p* = 0.570].

## Discussion

The present study used a data driven approach to explore IAPF variability in a group of university ice-hockey players at baseline and pre- and post-shooting task. Several studies within the cognitive domain have demonstrated that IAPF values are related to latent cognitive general factors (e.g., Grandy et al., 2013a), are strongly related to hereditability (e.g., Smit et al., 2006) and are not modifiable (e.g., Grandy et al., 2013b). These studies may partially explain the lack of variation in IAPF both at baseline, and pre-and post-task among groups.

Study results indicated no significant difference between baseline IAPF and performance groups (top, moderate, and bottom performers). Similarly, Angelakis et al. (2004b) did not find a relationship between IAPF (defined as PAF in their study) and cognitive performance in initial resting conditions. Rather, they demonstrated that IAPF after task in resting condition might reflect general cognitive ability (cognitive preparedness). Further, although IAPF is related to cognitive performance, it simply may not be related to motor performance ability for the specific task in this study. Hülsdünker et al. (2016) found significant differences in IAPF during a motor activity (balance tasks), however, IAPF was measured during the task itself rather than at eyes-closed resting condition pre-task. In the present study, IAPF was measured at rest and not during actual performance, thus future research could explore IAPF during the sport specific task itself.

As well, it is also possible that the sample itself needed greater diversity in performance ability in order to uncover significant differences. Although groups (top, moderate, and bottom performers) were statistically different based on performance scores (successful shots out of 50), all athletes, male and female, were playing at a university level and therefore they could be considered of similar ability. It is therefore suggested that future research should consider the same question but with an increased difference in ability levels, as in expert versus non-expert athletes.

It is also possible that there will be no relationship between IAPF and sport-performance ability if trait IAPF is in fact solely related to cognitive factors in a resting state. However, if the IAPF is related to ‘cognitive preparedness’ for a task, it may be prudent to revisit the methodology and revise instructions given to the participants prior to measurement. In EEG baseline measurements, participants were indeed asked to relax and close their eyes, and this is not necessarily how athletes *prepare* mentally for performance. Therefore, it might be useful in future research to instruct athlete-participants to close their eyes and prepare mentally for the task as they would prior to competition. Previous research has indicated that athlete mental preparation (i.e., imagery, self-talk) has a significant effect on performance (e.g., Gould et al., 2002; Bertollo et al., 2009). In sport, inconsistent mental preparation often leads to inconsistent performance, and therefore, how the athlete prepares pre-task may have a significant effect on the actual performance. Similar to Angelakis and colleagues’ results stating that pre-task IAPF was “significantly correlated with Digit Span performance of the same day but not with Digit Span performance of another day,” (Angelakis et al., 2004a, p. 887) the concept of cognitive preparedness for a specific task may be very applicable in the sport setting.

Further analysis of IAPF pre- and post-shooting task was completed in order to explore intra-subject variability in IAPF. Although IAPF slightly decreased for top performers and increased for moderate and bottom performers, there was no significant difference found for any of the groups. It has been suggested that a shift in IAFP occurs only when strong effort and cardio vascular and metabolic processes are involved and can be a marker of acute and chronic mental and physical fatigue (e.g., Billiot et al., 1997; Ng and Raveendran, 2007; Gutmann et al., 2015). Further, Gutmann et al. (2015) found that only exhaustive exercise caused a significant positive shift in IAPF compared to steady state exercise. The low complexity of the ice hockey shooting task itself may have attenuated cognitive demand, and therefore, is another factor to take into account in the interpretation of the data. Thus, it is possible that the shooting-task itself was not physically or cognitively demanding enough to show differences in IAPF.

In relation to cognitive preparedness, the study by Angelakis et al. (2004a) demonstrated a positive shift (increase) in IAPF in eyes-closed post-task resting condition for both high-IAPF (those whose IAPF was higher than the sample median) and low-IAPF (those whose IAPF was lower than the sample median) groups. The increase was only significant for the low-IAPF group suggesting that performing the task itself affects IAPF, and in this case, that subjects not fully prepared for the task (indicated by the low-IAPF initially) were “forced by the task to correct for their unpreparedness by increasing their [APF]” (Angelakis et al., 2004a, p. 894). The present data, although not significant, tentatively supports this suggestion by showing that both moderate and bottom performers increased their APF more than the top performers whose IAPF actually decreased. A separate analysis with the current data exploring the shift in IAPF between high- and low-IAPF groups revealed a slight increase in the low-IPAF group and a slight decrease in the high-IAPF group. Although not significant, this trend supports Angelakis and colleagues’ research and warrants further study. It is recommended that future research in the sport domain explore the correlation between IAPF at baseline (*ensuring proper sport-specific mental preparation)* during and after sport performance, evaluating day-by-day variation in IAPF. This analysis may help to better understand (a) whether cognitive preparedness (reflected in baseline IAPF) changes day-to-day or moment-to-moment prior to sport performance, and (b) whether these moment-to-moment changes are linked to performance in sport task.

This study also provides insight regarding the relevance of IAPF to the applied sport psychology domain. In general, neurofeedback training (NFT) in sport has been conducted with fixed frequency bands (i.e., low-alpha 8–10Hz and high-alpha 10–12Hz), which does not take into account the intra-individual differences in IAPF. Proper identification of IAPF and correlating individual alpha bands may be important in ensuring effective NFT. In fact, it has been suggested that knowing the inter- and intra-individual ranges of posterior alpha rhythm is “crucial in order to interpret results from studies that try to explain performance differences in terms of alpha activity modulations” (Haegens et al., 2014, p. 47). From an applied sport psychology perspective, further exploration of the link between IAPF and cognitive preparedness for a specific sport task could be beneficial in developing and training pre-performance routines for athletes in order to optimize performance. Specifically, NFT could be used as a tool to both identify and develop idiosyncratic peak mental readiness protocols for each athlete’s specific sport task. It has also been suggested that because IAPF increases with simple engagement in cognitive tasks, individuals could boost their level of cognitive preparedness through cognitive exercises or via NFT (Angelakis et al., 2004a). Notably, it has been shown that IAPF is linked to efficiency of NFT (Bazanova et al., 2009; Alekseeva et al., 2012) and therefore a shift in baseline IAPF may be a viable marker in determining the effectiveness of NFT training.

## Conclusion

Following a data driven approach to explore IAPF in ice hockey performance, this study extended research on IAPF in the cognitive domain to IAPF in the sport domain. The present study supports previous literature suggesting IAPF “is highly stable in healthy adults up to 80 years, not easily modifiable by cognitive interventions alone, and thus qualifies as a stable neurophysiological trait marker” (Grandy et al., 2013b, p. 570). Moreover, the results are not in contrast with preliminary findings in sport that demonstrated a shift in IAPF may occur with physical activity when strong effort and cardio vascular and metabolic processes are involved. Important methodological considerations should be kept in mind for exploring IAPF in sport: (a) measuring IAPF during sport performance instead of at a resting state, (b) changing the pre-performance resting baseline instructions to take into account sport-specific mental preparation, (c) exploring an expert-novice paradigm to accentuate performance ability differences between groups, (d) comparing tasks with different level of complexity, and (e) analyzing the possible correlation between IAPF and performance on different days.

## Author Contributions

SC: Responsible for conception and design of study. Developed the experimental design, conducted the experiments, analyzed and interpreted the data. Prepared and revised the final manuscript. Final approval of the version to be published. SdF: Assisted with data analysis. Provided critical revisions to the manuscript. Final approval of the version to be published. MB: Collaborated on conception and design of study. Consulted on every step of the analysis process. Supported analysis and interpretation of the data. Provided critical revisions to the manuscript. Final approval of the version to be published. PW: Collaborated on conception and design of study. Discussed the analysis and interpretation of the results. Provided critical revisions to the manuscript. Final approval of the version to be published.

## Conflict of Interest Statement

The authors declare that the research was conducted in the absence of any commercial or financial relationships that could be construed as a potential conflict of interest.
